# The Action of D-Dopachrome Tautomerase as an Adipokine in Adipocyte Lipid Metabolism

**DOI:** 10.1371/journal.pone.0033402

**Published:** 2012-03-12

**Authors:** Takeo Iwata, Hisaaki Taniguchi, Masamichi Kuwajima, Takako Taniguchi, Yuko Okuda, Akiko Sukeno, Kyoko Ishimoto, Noriko Mizusawa, Katsuhiko Yoshimoto

**Affiliations:** 1 Department of Medical Pharmacology, Institute of Health Biosciences, The University of Tokushima Graduate School, Tokushima, Japan; 2 Division of Disease Proteomics, Institute for Enzyme Research, The University of Tokushima, Tokushima, Japan; 3 Department of Clinical Biology and Medicine, Institute of Health Biosciences, The University of Tokushima Graduate School, Tokushima, Japan; 4 Taijukai-Kaisei General Hospital, Sakaide, Japan; 5 Department of Orthodontics and Dentofacial Orthopedics, Institute of Health Biosciences, The University of Tokushima Graduate School, Tokushima, Japan; John Hopkins Bloomberg School of Public Health, United States of America

## Abstract

Adipose tissue is a critical exchange center for complex energy transactions involving triacylglycerol storage and release. It also has an active endocrine role, releasing various adipose-derived cytokines (adipokines) that participate in complex pathways to maintain metabolic and vascular health. Here, we found D-dopachrome tautomerase (DDT) as an adipokine secreted from human adipocytes by a proteomic approach. DDT mRNA levels in human adipocytes were negatively correlated with obesity-related clinical parameters such as BMI, and visceral and subcutaneous fat areas. Experiments using SGBS cells, a human preadipocyte cell line, revealed that DDT mRNA levels were increased in an adipocyte differentiation-dependent manner and DDT was secreted from adipocytes. In DDT knockdown adipocytes differentiated from SGBS cells that were infected with the adenovirus expressing shRNA against the DDT gene, mRNA levels of genes involved in both lipolysis and lipogenesis were slightly but significantly increased. Furthermore, we investigated AMP-activated protein kinase (AMPK) signaling, which phosphorylates and inactivates enzymes involved in lipid metabolism, including hormone-sensitive lipase (HSL) and acetyl-CoA carboxylase (ACC), in DDT knockdown adipocytes. The AMPK phosphorylation of HSL Ser-565 and ACC Ser-79 was inhibited in DDT knockdown cells and recovered in the cells treated with recombinant DDT (rDDT), suggesting that down-regulated DDT in adipocytes brings about a state of active lipid metabolism. Furthermore, administration of rDDT in db/db mice improved glucose intolerance and decreased serum free fatty acids levels. In the adipose tissue from rDDT-treated db/db mice, not only increased levels of HSL phosphorylated by AMPK, but also decreased levels of HSL phosphorylated by protein kinase A (PKA), which phosphorylates HSL to promote its activity, were observed. These results suggested that DDT acts on adipocytes to regulate lipid metabolism through AMPK and/or PKA pathway(s) and improves glucose intolerance caused by obesity.

## Introduction

Adipose tissue is a critical exchange center for complex energy transactions involving triacylglycerol storage and also an active endocrine organ secreting adipokines that participate in complex pathways to maintain metabolic and vascular health [1]. Adipokines impact multiple functions such as appetite and energy balance, immunity, insulin sensitivity, angiogenesis, blood pressure, and lipid metabolism, and most of the adipokines that accumulate in obese individuals contribute to the pathogenesis of obesity-related disorders such as type 2 diabetes and cardiovascular diseases [2]. The identification of novel adipokines will improve our understanding of the endocrine function of adipose tissue, providing novel molecular targets for the treatment of obesity and related diseases.

In this study, we identified D-dopachrome tautomerase (DDT) as a protein secreted from adipocytes differentiated from human primary preadipocytes. DDT has been identified as an enzyme that converts D-dopachrome by tautomerization and decarboxylation to 5,6-dihydroxyindole [3]. Because the presence of D-dopachrome is unlikely in mammals, the conversion of D-dopachrome could hardly be the true function of DDT. The human DDT consists of 118 amino acids without putative signal sequences at the N-terminus and is expressed in various organs including the liver, heart, lung, and pancreas [4]. In terms of sequences and gene structure, this protein is related to macrophage migration inhibitory factor (MIF) [5], a proinflammatory cytokine released from activated macrophages and other immune effector cells [6], [7]. Furthermore, MIF has also tautomerase activity converting D-dopachrome to another tautomer, 5,6-dihydroxyindole-2-carboxylic acid [8]. Despite these intriguing similarities to MIF, there have been few studies on the biological functions of DDT. Although DDT was recently reported to be detectable in human serum and to function as a MIF homolog in non-small cell lung carcinomas and macrophages [9], [10], functions of DDT in other tissues are largely unknown.

White adipocytes store dietary energy as triacylglycerol through lipid synthesis and supply the free fatty acids (FFA) and glycerol generated by hydrolysing triacylglycerol to other organs in times of caloric need [1]. A breakdown in the regulation of adipocyte lipid metabolism can contribute to increased levels of FFA in the circulation, which is an established risk factor for the development of insulin resistance in type 2 diabetes and related disorders [11]. AMP-activated protein kinase (AMPK) is a major regulator of the cell's and whole body's metabolism [12]. The AMPK complex is an evolutionally conserved serine/threonine heterotrimer kinase complex consisting of α-, β- and γ-subunits [12]. Activation of AMPK is mediated through its major regulatory phosphorylation site Thr-172, located within the activation loop on the α-subunit [13]. Phosphorylation of this site in AMPK is essential for its activity and can be catalyzed by LKB or a Ca^2+^/calmodulin-dependent protein kinase, CaMKKβ [11]. On the other hand, protein kinase A (PKA) phosphorylates AMPK at Ser-173, 485, and 497 to inactivate AMPK [14], [15]. AMPK stimulates pathways which increase energy production (glucose transport and fatty acid oxidation), and switches off pathways which consume energy (lipogenesis, lipolysis, protein synthesis, and gluconeogenesis) [16]. AMPK inhibits lipogenesis and increases lipid oxidation mediated by a decrease in malonyl-CoA content due to inhibition of acetyl-CoA carboxylase (ACC) activity by phosphorylating its Ser-79 [17], whereas AMPK is known to prevent lipolysis by inhibiting hormone-sensitive lipase (HSL) activity by phosphorylating its Ser-565 [18]. These effects of AMPK tend to limit the release of free fatty acids (FFA) into plasma from adipocytes. Because FFA has a key role in the onset of insulin resistance, inactivating AMPK in adipose tissue may be a cause of insulin resistance. In this study, we investigated the phosphorylation of AMPK's substrates in DDT knockdown adipocytes and whether DDT improves glucose tolerance using db/db mice as a genetic model of obesity-associated insulin resistance.

## Materials and Methods

### Ethics statement

The study was approved by the Research Ethics Committee of the Tokushima University Hospital (approval number: 288) and written informed consent was obtained from each patient prior to their participation. The study was also conducted in accordance with the provisions of the Declaration of Helsinki. All animal experiments were approved by the animal research committee of the University of Tokushima (approval number: 11010).

### Antibodies

The rabbit polyclonal antibody against human recombinant DDT (rDDT) was made by Scrum Inc. (Tokyo, Japan). Antibodies against β-actin were purchased from Sigma (St. Louis, MO, USA). The antibody against fatty acid binding protein-4 (FABP4) was purchased from R&D Systems (Minneapolis, MN, USA). Antibodies against AMPKα, each phospho-AMPKα (Thr-172 and Ser-485), HSL, each phospho-HSL (Ser-565 and Ser-660), ACC, phospho-ACC (Ser-79), phospho-PKA substrate, and α/β tubulin were obtained from Cell Signaling (Beverly, CA, USA).

### Subjects

Nineteen subjects with various tumors participated. Paired visceral (the omentum) and subcutaneous (the abdominal wall) adipose tissues were biopsied from these subjects during the resection of tumors in the abdomen. Adipose tissue areas at the umbilical level in abdominal computed tomography were determined using commercially available software (Fat Scan; N2 System, Osaka, Japan). Human adipocytes and stromal vascular fraction (SVF) cells were fractionated from subcutaneous adipose tissues as described previously [19].

### Preparation of adenoviruses

The cDNA encoding short hairpin RNA (shRNA) against the DDT gene (GenBank accession number NM_001355.3) (shDDT) and nontargeting control shRNA (shNC) were purchased from Takara (Takara ID: TA0504-1 and TA0504-SN1, respectively; Shiga, Japan). The recombinant adenoviruses expressing shRNA were constructed using an Adenovirus Expression Vector Kit (Takara) according to the manufacturer's instructions. For the construction of adenovirus expressing DDT-FLAG, cDNA encoding human DDT with the FLAG sequence at the C-terminus, was inserted into the pAxCAwtit and the adenovirus was produced in the same manner. The adenovirus expressingβ-galactosidase was constructed from a control cosmid pAxCAiLacZit supplied with the Kit. Viral titers were determined using the AdenoX rapid titer assay kit (Clontech, Mountain View, CA, USA).

### Cells

SGBS cells, a human preadipocyte cell line, were maintained and made to differentiate into adipocytes as described by Wabitsch *et al*. [20]. Confluent SGBS cells were transduced with each adenovirus at a multiplicity of infection of 100 and subjected to adipogenic induction 24 h after the infection. At 9 days after adipogenic induction, the cells were treated with 2 nM rDDT for 24 h or 5-aminoimidazole-4-carboxamide-1-β-D-ribonucleotide (AICAR; Sigma) for 30 min.

### Reverse transcription-quantitative polymerase chain reaction (RT-qPCR)

Total RNA was isolated using ISOGEN (Nippongene, Tokyo, Japan), according to manufacture's instruction and quantified by measuring absorbance at 260 nm. The ratio of absorbance at 260 nm to that at 280 nm in total RNA was between 1.9 and 2.0. RNA integrity was further assessed by electrophoresis on a formaldehyde agarose gel. cDNA was synthesized from 500 ng RNA with a mixture of oligo-dT primers and random oligomers in a 20 µl reaction volume using a PrimeScript™ RT Reagent Kit (Takara), according to manufacture's instruction. Then, each sample cDNA and a standard cDNA selected from samples were diluted 10-fold and 2-fold with TE buffer (10 mM Tris, 0.1 mM EDTA, pH 8.0), respectively. The standard cDNA was further subjected to serial 10-fold dilutions to calculate the standard curve and to measure the amplification efficiency for each target and reference gene. cDNA obtained was immediately used for qPCR analysis.

RT-qPCR analysis were carried out in a 7300 Real time PCR System (Applied Biosystems, Foster City, CA) according to the following program: 10 min at 95°C, followed by 45 cycles of 95°C for 15 sec and 60°C for 1 min and additional cycle of dissociation curves to ensure an unique amplification. The reaction mixture in a final volume of 10 µl contained 1 µl of cDNA, 5 µl of THUNDERBIRD SYBR qPCR mix (Toyobo, Osaka, Japan), 0.2 µl of ROX reference dye, and 1 µM of each gene-specific primer set designed to bind to exons flanking an intron, except for leptin and C/EBPα genes (Table 1). At least duplicate reactions for each sample cDNA, the standard cDNAs, and no template controls (NTC) using the same primer set were analyzed together in the same optical 96-well plate. Additionally 50 ng of RNA from each sample without RT was used in the qPCR to assess genomic DNA contamination. The specificity of the amplification products was confirmed by size estimations on a 2% agarose gel and analyzing their melting curves. The data were analyzed by the relative standard curve method using 7300 system sequence detection software (version 3.1; Applied Biosystems) to determine the relative quantitative gene expression. Slopes in standard samples for the target genes were confirmed to lie between −3.6 and −3.2 and the each R2 value was between 0.99 and 1. Crossing points (Cq) were determined by setting the threshold automatic. All Cq for the target genes in samples were within range of the standard curves and were less than 35 cycles. On the other hand, NTC and no RT controls did not reach the threshold. The relative mRNA level of each target gene was normalized using human glyceraldehyde 3-phosphate dehydrogenase (GAPDH) or mouse β-actin as a reference gene, whose expression was confirmed not to be changed during adipogenesis in SGBS cells, or not to differ between adipocytes from db/db and wild-type mice (data not shown). Furthermore, we also obtained the same results using other reference genes such as TATA binding protein, hypoxanthine phosphoribosyltransferase 1, and 18S ribsomal RNA.

**Table 1:** Nucleotide sequences of primers used for RT-qPCR.

Gene		Primer Sequence
human ACC1	F	5′-TGATGTCAATCTCCCCGCAGC-3′
(NM_198834.1)	R	5′-TTGCTTCTTCTCTGTTTTCTCCCC-3′
human Adiponectin	F	5′-GTGATGGCAGAGATGGCAC-3′
(NM_001177800.1)	R	5′-ACACTGAATGCTGAGCGGTA-3′
human AEBP1	F	5′-ATGGGTGATGTACACCAACGGCTA-3′
(NM_001129.3)	R	5′-AGTGGGTAGATGCGGATGAAACGA-3′
human ATGL	F	5′-GTGTCAGACGGCGAGAATG-3′
(NM_020376.3)	R	5′-TGGAGGGAGGGAGGGATG-3′
human C/EBPα	F	5′-AAGAAGTCGGTGGACAAGAACAG-3′
(NM_004364.3)	R	5′-GCAGGCGGTCATTGTCACT-3′
human DGAT2	F	5′-CTCTTCTCCTCCGACACCTG-3′
(NM_032564.3)	R	5′-TGGTCTTGTGCTTGTCGAAG-3′
human DDT	F	5′-CGCCCACTTCTTTGAGTTTC-3′
(NM_001355.3)	R	5′-GGAAGAAGCAGCCAGTTCAC-3′
human FABP4	F	5′-CCTGGTACATGTGCAGAAAT-3′
(NM_001442.2)	R	5′-AGAGTTCAATGCGAACTTCA-3′
human GAPDH	F	5′-GAAGGTGAAGGTCGGAGTC-3′
(NM_002046.3)	R	5′-GAAGATGGTGATGGGATTTC-3′
human GPAM	F	5′-CCAGCCTGTGCTACCTTCTC-3′
(NM_020918.4)	R	5′-GAAGCTTCTTGTCCCACTGC-3′
human HSL	F	5′-ACTGCCAGCTGCCTTAAAAA-3′
(NM_005357.2)	R	5′-CCTCTGGTGTGGTTCAGGTT-3′
human Leptin	F	5′-AGAAAGTCCAGGATGACACC-3′
(NM_000230.2)	R	5′-GACTGCGTGTGTGAAATGTC-3′
human PLIN	F	5′-CTCTCGATACACCGTGCAGA-3′
(NM_001145311.1)	R	5′-TGGTCCTCATGATCCTCCTC-3′
human PPARγ2	F	5′-TCCATGCTGTTATGGGTGAA-3′
(NM_015869.4)	R	5′-CAAAGGAGTGGGAGTGGTC-3′
mouse β-actin	F	5′-GGCTGTATTCCCCTCCATCG-3′
(NM_007393.3)	R	5′-CCAGTTGGTAACAATGCCATGT-3′
mouse Ddt	F	5′-CTCTTCTCCCGCTAACATGC-3′
(NM_010027.1)	R	5′-TCATGCCAGGTCGTATCGTA-3′

The GenBank accession number is indicated below each gene.

### Immunofluorescent staining

The cells were fixed in 10% formaldehyde and then incubated in phosphate-buffered saline (PBS) with 0.1% Triton X-100 for 10 min followed by blocking solution (Blocking One: Nacalai Tesque, Kyoto, Japan) for 1 h. The cells were incubated with a 1∶100 dilution of rabbit anti-DDT antibody, or 1∶100 dilution of normal rabbit IgG for 2 h, and then incubated with a 1∶500 dilution of Alexa Fluor 488 goat anti-rabbit IgG (Invitrogen) including 4,6-diamidino-2-phenylindole (DAPI; Sigma) for 30 min at 37°C. Next, they were stained with a 0.3% Sudan III solution. The cells were observed under a fluorescence microscope (Nikon Eclipse TE2000U microscope; Nikon, Tokyo, Japan) and images were obtained using DIGITAL SIGHT DS-L1 (Nikon).

### Western blot analysis

To obtain conditioned medium (CM), the cells on 6-well plates were washed with PBS and cultured in 1 ml/well of fresh serum-free medium for 24 h. The proteins in the 5 ml of CM were precipitated with a 4-fold volume of cold acetone and the precipitation was dissolved in 100 µl of PBS. Samples of the cells or adipose tissue were lysed with lysis buffer (50 mM Tris-Cl, 150 mM NaCl, and 0.5% NP-40, pH 8.0, supplemented with complete protease inhibitor cocktail (Roche; Basel, Switzerland)). The protein samples were subjected to SDS-PAGE and transferred onto Immobilon Transfer Membranes (Millipore, Bedford, MA, USA). Membranes were blocked with Blocking One and probed with each primary antibody followed by horseradish peroxidase-conjugated secondary antibody (GE Healthcare, Buckinghamshire, UK). Antigens were then visualized by enhanced chemiluminescence (Immobilon Western; Millipore) using X-ray film. The optical density of the protein band was calculated by Image J software (National Institute of Health, Bethesda, MD, USA).

### Preparation of rDDT

Human DDT cDNA was inserted into the pGEX 6p-3 vector (GE Healthcare). The clone was introduced into competent BL21 (DE3) cells (Takara) and expression of the DDT fused to glutathione-S-transferase (DDT-GST) was induced by 1 mM isopropyl β-D-thiogalactoside (WAKO) for 16 h at 18°C. The bacteria were lysed by mild sonication at 4°C in sonication buffer (50 mM Tris, pH 8.0, 50 mM NaCl, and 1 mM ethylene diamine tetraacetic acid) supplemented with Complete Mini (Roche). DDT-GST was affinity-purified from clarified lysate using glutahione-S-sepharose gels, and GST was removed by digestion with Precission protease (Roche) at 4°C. Then, rDDT was fractionated by chromatography with a Mono Q column (GE Healthcare) and Sepharose G-50 column (GE Healthcare). After desalting by a Centriplus YM-3K (Millipore), rDDT was prepared at concentration of 60 µM in PBS.

### Animal studies

We used 7-week-old male BKS.Cg-m+/+ (control mice) and BKS.Cg-m+/+ Lepr^db^ (db/db mice) obtained from Japan CLEA (Tokyo, Japan). Mice were injected intraperitoneally with rDDT (100 nmol/kg body weight) seven times in 2-day intervals. For a glucose tolerance test (GTT), mice were injected intraperitoneally with glucose (2 g/kg body weight) after a 16-h fast. An insulin tolerance test (ITT) was carried out by intraperitoneal injection of insulin (0.75 IU/kg body weight) to mice after a 6-h fast. Blood glucose levels were measured by Glucocard Diameter (Arkray, Kyoto, Japan). Blood samples from a tail vein and epididymal adipose tissue were collected from mice 1 h after the last injection under anesthesia. Serum insulin and FFA levels were measured by Rebis Insulin Mouse T (Shibayagi, Gunma, Japan) and Free fatty acids, Half-micro test (Roche), respectively.

### Statistical analysis

The correlation among DDT mRNA levels and various parameters was assessed by Pearson's method with SPSS14.0 for Windows (SPSS Inc., Chicago, IL, USA). Statistical analyses were performed using Student's *t*-test. Differences were considered to be significant when the *P*-value was less than 0.05.

## Results

### DDT as a protein secreted from adipocytes and its correlation with obesity

**Figure 1:**
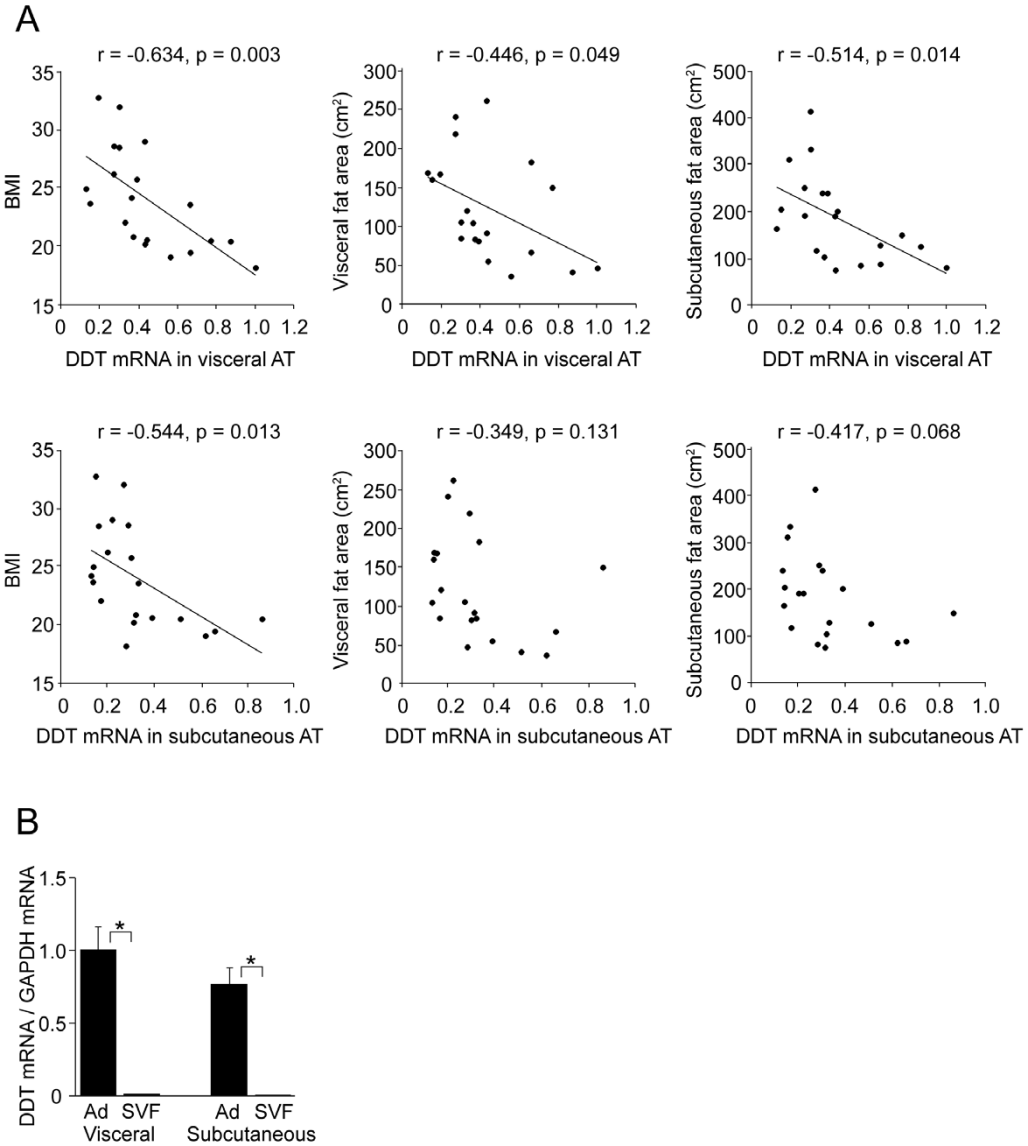
DDT mRNA expression in the adipose tissues. **A** Correlation between DDT mRNA levels in adipocytes fractionated from visceral and subcutaneous adipose tissues (AT) and each obesity-related clinical parameter in 19 subjects. The normalized DDT mRNA levels are blotted relative to the highest level of DDT mRNA in visceral AT from a patient. **B** DDT mRNA levels in adipocytes (Ad) and SVF cells (SVF) fractionated from visceral and subcutaneous AT of 19 patients. The normalized DDT mRNA levels are shown relative to those in visceral adipocytes. Data are the mean ± SEM (n = 19). *P<0.05.

To identify proteins secreted from human adipocytes, we performed a proteomic analysis of the CM of adipocytes differentiated from human preadipocytes (Taniguchi *et al*, in preparations). To screen for noteworthy proteins among adipokine-candidates, correlations between the mRNA levels of each protein in adipocytes and obesity-related clinical parameters were examined. Among these candidates, we focused on DDT, because its mRNA levels in adipocytes fractionated from visceral adipose tissue showed a negative correlation with body mass index (BMI), or visceral or subcutaneous fat area (Fig. 1A). Although DDT mRNA levels in subcutaneous adipose tissues did not show a significant correlation with each fat area, they tended to be low in obese samples. DDT mRNA expression was observed in mature adipocytes, but not SVF cells, and did not differ significantly between adipocytes derived from visceral and subcutaneous adipose tissues (Fig. 1B).

### DDT is expressed in adipocytes but not preadipocytes

**Figure 2:**
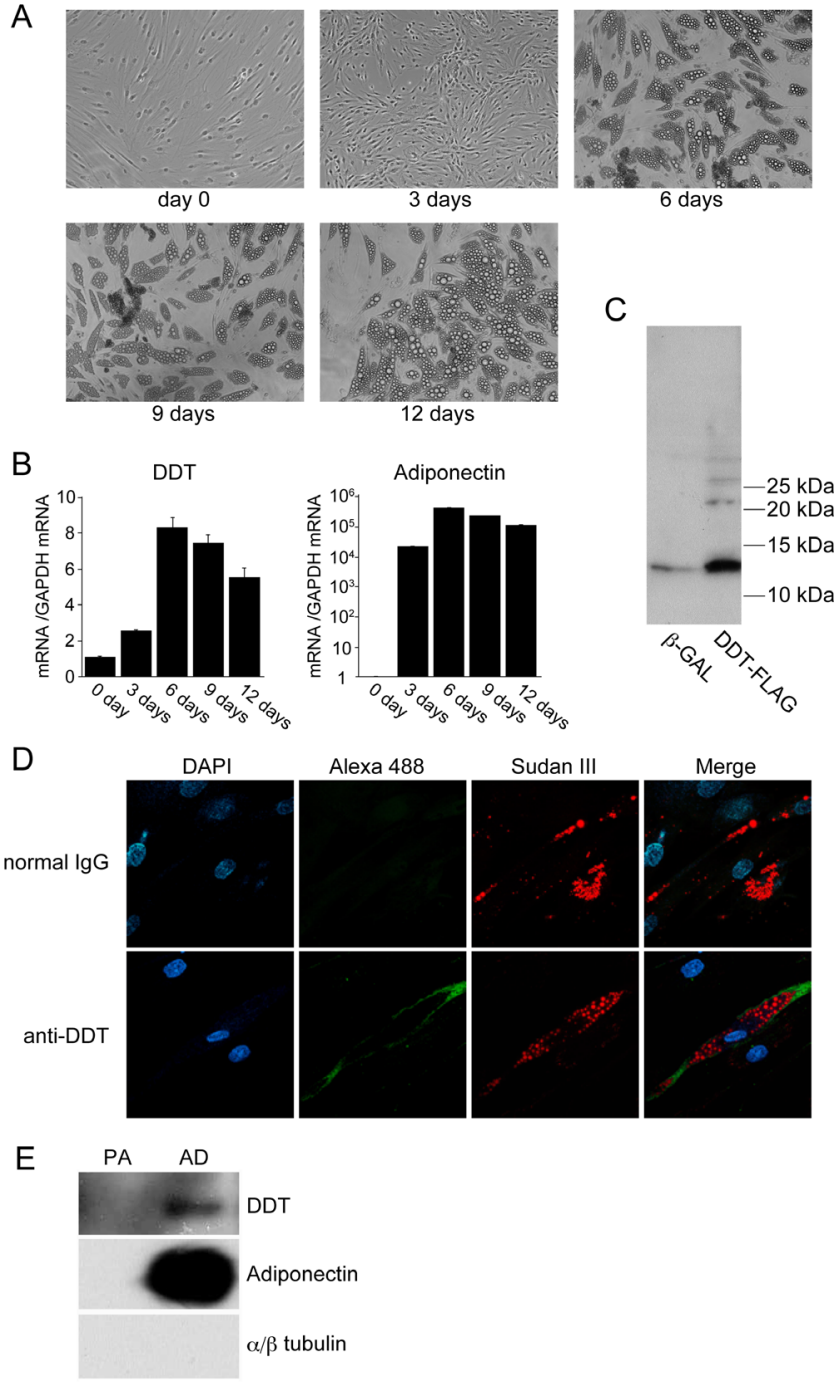
DDT expression in adipocytes and preadipocytes. **A** Development of lipid droplets in differentiated adipocytes from SGBS cells. No lipid droplets were observed in confluent SGBS cells (day 0). After differentiation was induced, lipid droplets started appearing at 6 days, and were enlarged by 9 days and 12 days. **B** DDT and adiponectin mRNA levels during differentiation of SGBS cells into adipocytes. The normalized mRNA levels are shown relative to those at day 0. The graph is representative of 3 independent experiments. Data are the mean ± SEM (n = 3). **C** Detection of endogenous DDT in adipocytes using an anti-DDT antibody. Lysates of adipocytes derived from SGBS cells infected with the adenovirus expressing β-gal (β-GAL) or FLAG tagged DDT (DDT-FLAG) were subjected to SDS-PAGE and Western blotting using the anti-DDT antibody. **D** Immuncytochemistry using the anti-DDT antibody or normal rabbit IgG as a negative control in SGBS adipocytes. DAPI (blue); DDT (Alexa 488, green); Sudan III (red). Endogenous DDT was detected only in cells with lipid droplets. **E** Secretion of DDT from adipocytes. Western blot analysis of the concentrated CM (fifty times) from SGBS preadipocytes (PA) and adipocytes differentiated from SGBS cells (AD) was performed.

We investigated the difference in DDT mRNA expression between preadipocytes and mature adipocytes using human preadipocyte cell line, SGBS cells, which can efficiently and reproducibly be induced to develop into adipocytes [20], because each preadipocyte from patient's adipose tissue had different adipogenic efficiency among individuals or the passage numbers. DDT mRNA levels were measured during the course of differentiation in SGBS cells. Lipid droplets were observed in the cells from 3 days after adipogenic induction and gradually increased in size (Fig. 2A). As shown in Fig. 2B, DDT mRNA levels were increased from days 3 to 6, and gradually decreased at days 9–12 as did adiponectin mRNA levels, indicating that DDT mRNA expression is induced during adipogenesis. To detect endogenous DDT expression, we made a rabbit polyclonal antibody against human rDDT. Western blot analysis of cell lysate from adipocytes infected with the adenovirus expressing DDT or β-galactosidase revealed that this antibody could detect both endogenous and exogenous DDT of approximately 13 kDa (Fig. 2C). Immunofluorescent study showed DDT expression in the cytoplasm in differentiated adipocytes, but not in cells without lipid droplets (Fig. 2D), indicating that DDT is expressed in mature adipocytes but not preadipocytes. To confirm its secretion from adipocytes, the CM of adipocytes differentiated from SGBS cells was concentrated to fifty times and then subjected to Western blot analysis using DDT antibody. DDT was detected with signals lower than adiponectin in the concentrated CM of differentiated adipocytes, but not undifferentiated SGBS cells (Fig. 2E). The concentrated CM did not include α/β tubulin, suggesting no contamination of cytosolic contents in the CM. This result suggested that DDT is a protein secreted from adipocytes, although at concentrations far lower than adiponectin.

### DDT knockdown influences expression of genes involved in lipid metabolism

**Figure 3:**
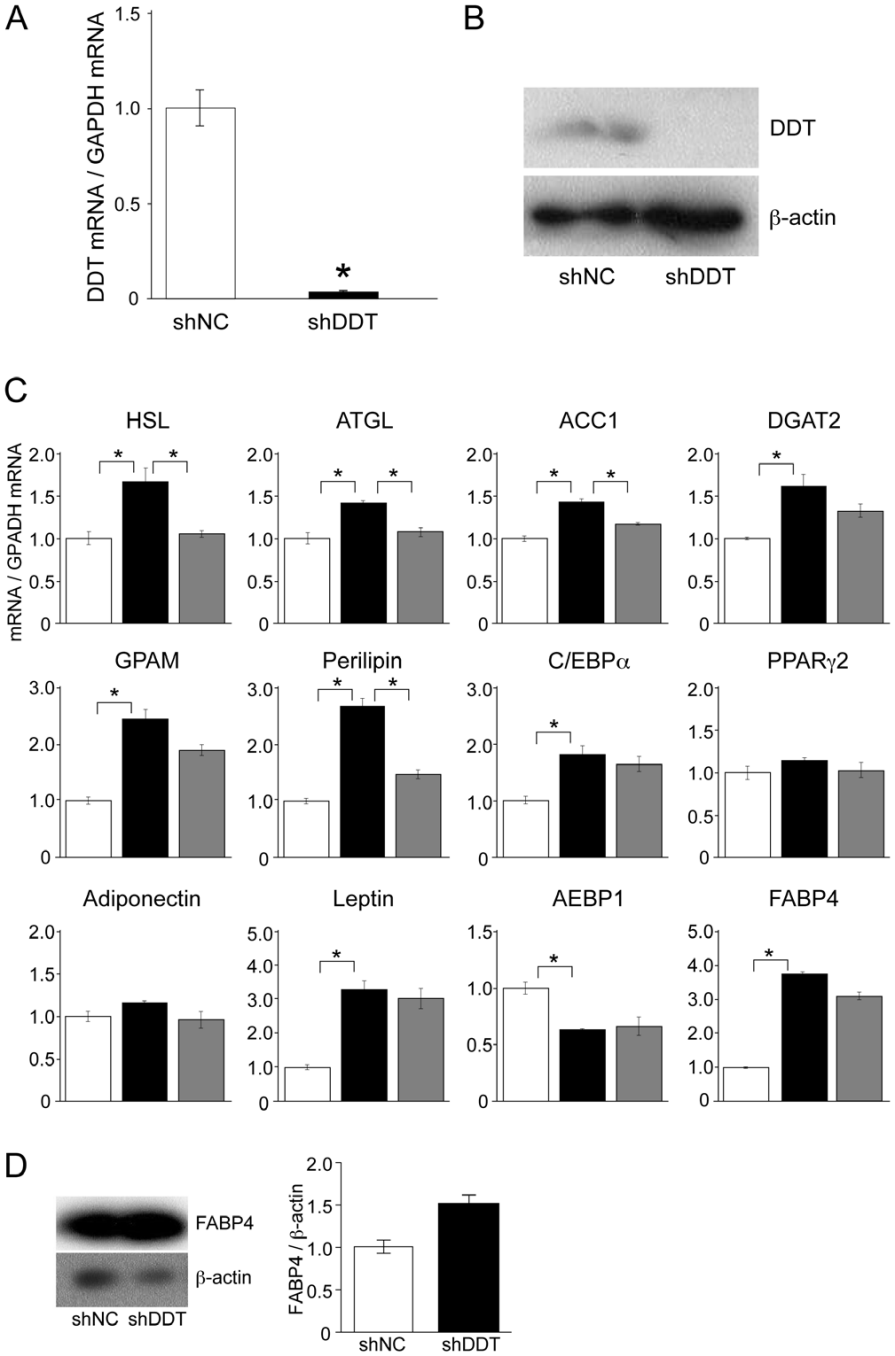
Effects of DDT knockdown on the expression of genes related to lipid metabolism. **A, B** DDT mRNA (**A**) and protein (**B**) expression in adipocytes differentiated from SGBS cells infected with adenovirus expressing shDDT or shNC. mRNA and protein levels were detected by RT-qPCR and Western blotting using anti-DDT antibody, respectively. The normalized DDT mRNA levels are shown relative to those in cells expressing shNC. The graph is representative of 5 independent experiments. Data are the mean ± SEM (n = 3). *P<0.05. **C** At 9 days after adipogenic induction, total RNA was extracted from cells subjected to infection with the adenovirus expressing shNC (white bars) or shDDT (black bars). DDT knockdown adipocytes were treated with 2 nM rDDT for 24 h and total RNA was extracted (gray bars). The mRNA levels of lipid metabolism-related genes in the cells were measured by RT-qPCR. The expression of each gene was normalized to that of GAPDH mRNA. The normalized DDT mRNA levels are shown relative to those in cells expressing shNC. The graph is representative of 5 independent experiments. Data are the mean ± SEM (n = 3). *P<0.05. **D** FABP4 protein expression in adipocytes. Western blot analysis of lysate prepared from cells infected with the adenovirus expressing shNC or shDDT was performed with an FABP4 antibody. The graph represents band density ratio of FABP4 to β-actin. The levels are shown relative to those in cells expressing shNC. The graph is representative of 3 independent experiments. Data are the mean ± SEM (n = 3). *P<0.05.

To examine the function of DDT in adipocytes, we produced DDT knockdown adipocytes using the adenovirus expressing shDDT. In DDT knockdown adipocytes, a reduction in both mRNA and protein levels of DDT compared with the adipocytes infected with the adenovirus expressing shNC was confirmed (Fig. 3A, B). DDT knockdown slightly but significantly increased the expression of genes involved in lipolysis (HSL, adipocyte triglyceride lipase; ATGL, and perilipin) and lipogenesis (ACC1, diacylglycerol O-acetyltransferase-2; DGAT2, glycerol-3-phosphate acyltransferase 1, mitochondrial; GPAM) (Fig. 3C). Furthermore, mRNA levels of CAAT/enhancer binding protein α (C/EBPα), a master regulator of adipogenesis, and leptin were significantly elevated, whereas mRNA levels of peroxisome proliferation-activated receptor γ 2 (PPARγ2), another master regulator of adipogenesis, and adiponectin were not. Interestingly, mRNA levels of FABP4 (also designated aP2 or AFABP), which was recently reported to associate with insulin resistance, and adipocyte enhancer binding protein 1 (AEBP1), a transcriptional repressor of the FABP4 gene, were remarkably higher and lower in the knockdown adipocytes, respectively. Protein levels of FABP4 were also increased (Fig. 3D).

To investigate whether the changes of gene expression were due to a lack of secretion of DDT, DDT knockdown cells were treated with rDDT (Fig. 3C, gray columns). The treatment significantly reduced the mRNA expression of HSL, ATGL, perilipin, and ACC1, suggesting that secreted DDT influences gene expression in adipocytes. The effective concentration (2 nM) of rDDT was consistent with that of experiments using macrophages by Merk *et al.* [10] and was within the physiologic range. On the other hand, rDDT-treatment had a partial rescuing effect on FABP4, AEBP1, and C/EBPα mRNA expression. These changes in mRNA levels may be caused by the function of intercellular DDT or by the chronic effect of DDT's depletion.

### DDT knockdown decreases the phosphorylation of HSL and ACC by AMPK

**Figure 4:**
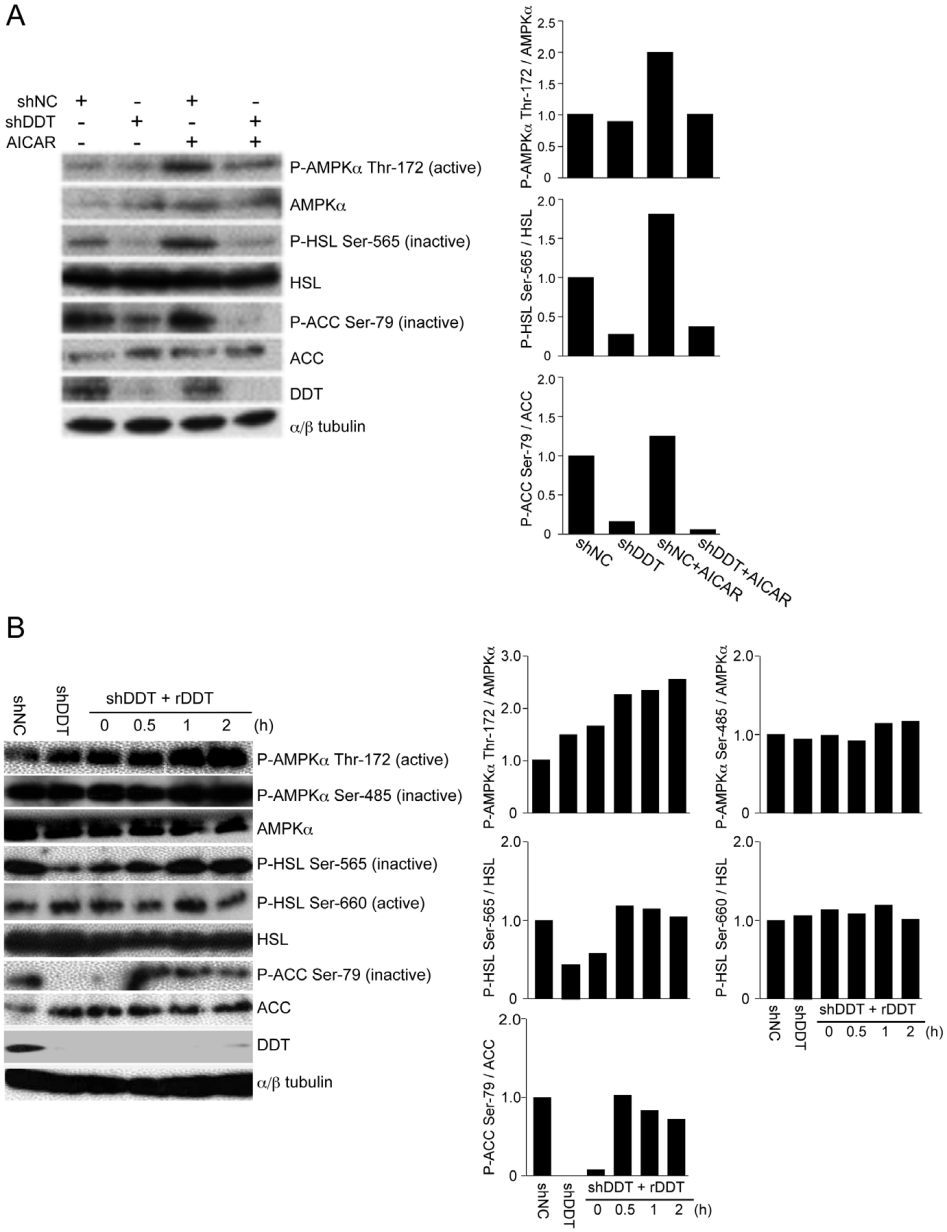
Effects of DDT knockdown on phosphorylation of AMPKα at Thr-172, HSL at Ser-565, and ACC at Ser-79. **A** At 9 days after adipogenic induction, adipocytes differentiated from SGBS cells infected with an adenovirus expressing shNC or shDDT were used. These cells were cultivated for 30 min in the presence or absence of AICAR. Protein lysate was prepared and probed with the indicated antibodies. An image shown is representative of 3 independent experiments. The graphs represents band density ratio of each phosphorylated protein to total protein in a left image. **B** Cells treated in the same manner as in (**a**) were used. The cells treated with 2 nM rDDT in serum-free medium were cultivated for the period indicated. Protein lysate was prepared and probed with the indicated antibodies. An image shown is representative of 3 independent experiments. The graphs represents band density ratio of each phosphorylated protein to total protein in a left image.

Based on the elevated expression of genes related to lipid metabolism in DDT knockdown adipocytes, we investigated the involvement of AMPK, a key enzyme regulating both lipolysis and lipogenesis in adipocytes. Unexpectedly, no apparent decrease in the basal level of AMPKα phosphorylated at Thr-172, indicating AMPK activation, in DDT knockdown cells was observed (Fig. 4A). However, phosphorylation of AMPKα at Thr-172 induced by an AMP-mimicking agent, AICAR, was inhibited in DDT knockdown cells. AICAR treatment also induced the phosphorylation of HSL at Ser-565 and ACC at Ser-79, which was catalyzed by AMPK to inhibit those activities, in adipocytes. Phosphorylation of HSL at Ser-565 and ACC at Ser-79 was clearly inhibited in DDT knockdown cells irrespective of AICAR treatment. These results suggested that HSL and ACC are activated in DDT knockdown adipocytes. Unlike the mRNA expression of HSL and ACC, the protein levels did not remarkably increase in DDT knockdown adipocytes. This may be derived from difference of sensitivity between RT-qPCR and western blot analysis, because changes of these mRNA levels in DDT knockdown were slight. Furthermore, treatment with rDDT for 30 min increased the phosphorylation of AMPKα at Thr-172, HSL at Ser-565, and ACC at Ser-79 in DDT knockdown adipocytes (Fig. 4B), suggesting that DDT acts on adipocytes to inhibit HSL and ACC activities by activating AMPK. Furthermore, we investigated involvement of PKA in AMPK and HSL phosphorylation, because PKA also plays an essential role in lipolysis mediating hormone-stimulated AMPK inactivation and HSL activation [14]. PKA directly phosphorylates HSL at Ser-563, 659, and 660, leading to translocation of HSL to lipid droplets, resulting in lipolysis and phosphorylates AMPK at Ser-173, 485, and 497 to inactivate AMPK [14], [15]. Treatment of rDDT did not affect the phosphorylation levels of AMPK at Ser-485 and HSL at Ser-660 in DDT knockdown adipocytes (Fig. 4B).

### rDDT administration improves glucose intolerance in db/db mice

**Figure 5:**
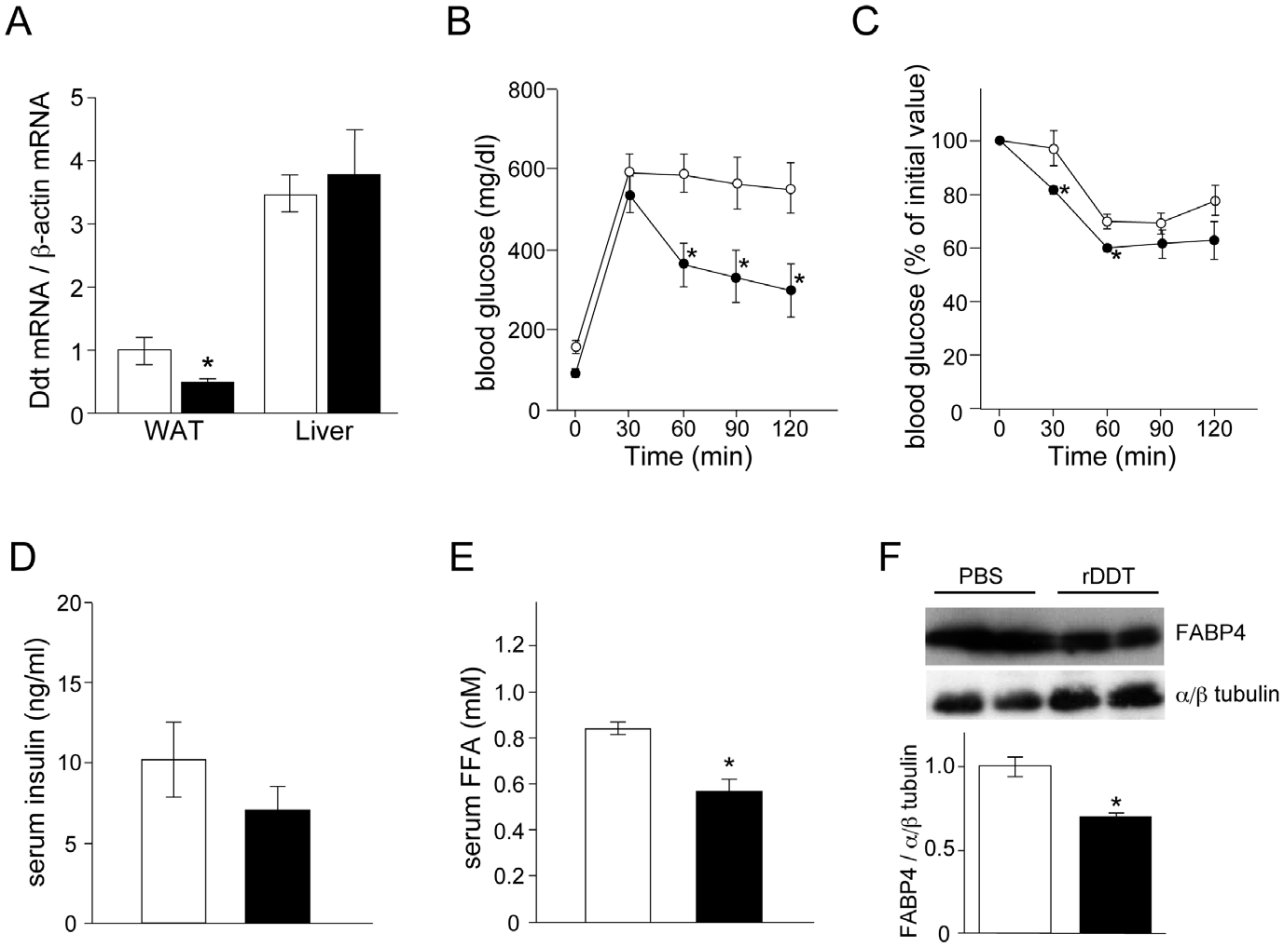
Effect of rDDT injection in db/db mice on glucose intolerance. **A** mouse DDT (Ddt) mRNA levels in the epididymal white adipose tissue (WAT) and liver from wild-type mice (white bars) and db/db mice (black bars). mRNA levels of Ddt were measured by RT-qPCR. The normalized Ddt mRNA levels are shown relative to those in WAT from wild-type mice. **B** A GTT in db/db mice injected with PBS (white circles) or rDDT (black circles) for 2 weeks. These mice fasted for 16 h were injected intraperitoneally with 2 g/kg of glucose and blood glucose was monitored at the indicated times. **C** An ITT in db/db mice treated with PBS (white circles) or rDDT (black circles). These mice fasted for 4 h was injected intraperitoneally with 0.75 IU/kg of insulin and blood glucose was monitored at the indicated times. **D, E** Fasting serum insulin (**D**) and FFA (**E**) levels in db/db mice treated with PBS (white bar) or rDDT (black bar). **F** FABP4 levels in the epididymal adipose tissue from db/db mice injected with PBS or rDDT. The WAT was removed from each mouse 1 h after the last PBS/rDDT injection and a Western blot analysis of FABP4 and ~αβ-tubulin was performed. The image is representative of 3 independent experiments. The graph represents mean of band density ratio of FABP4 to ~αβ tubulin in WAT from each mice. The levels are shown relative to those in WAT from wild-type mice. **A–F** Each graph is representative of 3 independent experiments. Data are the mean ± SEM (n = 4). *P<0.05.

The decrease in the phosphorylation of AMPK's substrates and increase of FABP4 expression in DDT knockdown adipocytes led us to speculate that the down-regulation of DDT expression is involved in glucose intolerance caused by obesity. Therefore, we examined effects of rDDT on insulin resistance in db/db mice. In db/db mice, Ddt mRNA levels in the adipose tissue, but not the liver, were lower than in the control mice (Fig. 5A), consistent with the results obtained *in vitro* with human adipocytes (Fig. 1A). Administration of rDDT for 2 weeks in db/db mice did not influence weight gain (data not shown). A GTT and an ITT showed that glucose intolerance in db/db mice was reversed by treatment with rDDT (Fig. 5B, C). Although serum insulin levels did not change significantly (Fig. 5D), serum FFA levels were significantly lower in rDDT-treated db/db mice (Fig. 5E). Furthermore, FABP4 expression in the adipose tissue was reduced by rDDT administration (Fig. 5F).

**Figure 6:**
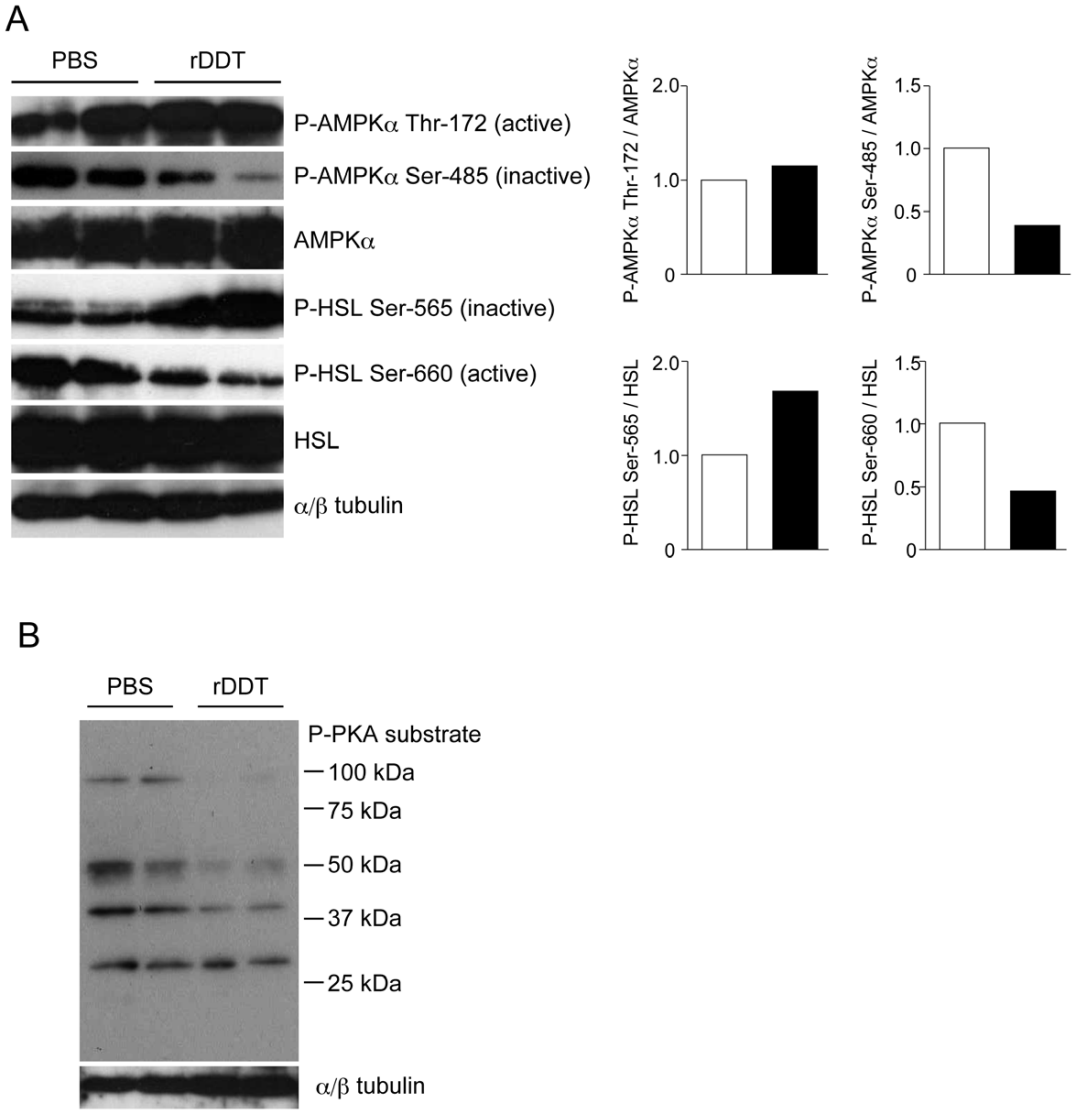
Phosphorylation of HSL, AMPK, and PKA's substrates in the adipose tissues from rDDT-treated db/db mice. **A** Phosphorylation of HSL and AMPK in the epididymal adipose tissues removed from each mouse 1 h after the injection of PBS or rDDT. Western blot analysis using indicated antibodies was performed. An image shown is representative of 3 independent experiments. The graph represents band density ratio of each phosphorylated protein to total protein obtained from a left image. White and black bars denote mice injected PBS and rDDT, respectively. The graph represents mean of band density ratio of each phosphorylated protein to total protein in the adipose tissue from db/db mice injected with PBS (white bars) or rDDT (black bars). **B** Phosphorylation of PKA's substrates in the epididymal adipose tissues removed from each mouse 1 h after the injection of PBS or rDDT. For detection of the phosphorylation of PKA's substrates, antibody recognizing phosphorylated Ser or Thr in the RRXS/T motif was used.

Finally, we investigated the phosphorylation of HSL in the epididymal adipose tissue from rDDT-treated db/db mice (Fig. 6A). Levels of HSL phosphorylated at Ser-565, which corresponds to reduced activity of HSL, were higher in rDDT-treated db/db mice, consistent with the *in vitro* data obtained from SGBS adipocytes (Fig. 4A, B). Although changes in levels of AMPKα phosphorylated at Thr-172 were not remarkable, levels of AMPKα phosphorylated at Ser-485 by PKA and involved in the negative regulation of AMPK, were lower in rDDT-treated db/db mice. This suggested that PKA was involved in the increased phosphorylation of AMPK's substrates by DDT *in vivo*. Furthermore, HSL Ser-660 phosphorylation with its higher activity, which was also phosphorylated by PKA, was inhibited in rDDT-treated db/db mice. Western blot analysis using a phospho-PKA substrate antibody revealed that PKA activity was decreased in the adipose tissue from rDDT-treated db/db mice (Fig. 6B).

## Discussion

DDT is expressed in various tissues including the liver, heart, lung, and pancreas [4]. DDT activity was reported to be increased in blister fluid following ultraviolet B irradiation [21]. In addition, its expression in the liver is induced by stimuli such as carbon tetrachloride, partial hepatectomy, and hepatitis B virus [22]–[24]. Thus, DDT levels vary with environments or stimuli, suggesting DDT to have certain physiological functions in tissues. We showed that levels of DDT mRNA in human adipocytes were negatively correlated with obesity-related parameters such as BMI or visceral and subcutaneous fat areas. We could not rule out the possibility that its expression was dependent on tumor status, because investigated adipocytes were derived from patients with various tumors. However, its lower levels in the adipose tissue from db/db mice also were consistent with the lower levels of DDT mRNA in adipocytes from obese patients. The reduction in obesity may be adipocyte-specific, because the mRNA levels in the liver did not differ between db/db mice and control mice. Furthermore, we showed that DDT is a protein secreted form adipocytes. The serum levels of DDT are probably extremely low, because we could not measure the serum levels or the amounts secreted from adipocytes by our enzyme-linked immunosorbent assay (detective range >100 ng/ml) using our anti-DDT antibody (data not shown). DDT is recently reported to be detectable in human serum and the median concentration is 6.9 ng/ml [10].

Based on the expression pattern, we investigated the physiological functions of DDT in adipocytes. We found increased levels of mRNA encoding proteins involved in lipolysis and lipogenesis in DDT knockdown adipocytes. Although elevation of these mRNA levels was almost slight, the results led us to build a hypothesis that DDT is involved in lipid metabolism in adipocytes. Indeed, we found the reductions in the basal phosphorylation of AMPK's substrates, HSL and ACC, in DDT knockdown adipocytes. This suggested that DDT-depletion in adipocytes brought about the activation of lipid metabolism by enhancing gene expression and inhibiting AMPK activity. The elevated mRNA levels of several genes in DDT knockdown adipocytes may be due to inactivation of AMPK, because activated AMPK inhibits the transcription of genes encoding lipogenic enzymes including ACC1 [25], [26]; however, the possibility that DDT regulates gene expression through mechanisms other than AMPK could not be excluded. Moreover, the administration of rDDT to DDT knockdown cells rescued the mRNA levels in several genes and phosphorylation levels of AMPK's substrates, indicating that DDT secreted from adipocytes acts on adipocytes in an autocrine or paracrine manner. Leptin and adiponectin are known to activate AMPK [27], [28]. Since these adipokines are highly expressed in adipocytes and their serum levels are high, their circulation affects various organs. On the other hand, amounts of DDT secreted from adipocytes are probably small as shown in Fig. 2E. Furthermore, rDDT administration to SGBS adipocytes expressing endogenous DDT did not induce gene expression or phosphorylation of AMPK's substrates (data not shown). Therefore, DDT may be effective to adipocytes at the concentration far less than adiponectin or leptin and normal adipocytes may secrete enough amount of DDT to retain AMPK activity at levels necessary for maintaining basal lipid metabolism.

FABP4 mRNA and protein levels were higher in DDT knockdown adipocytes. FABP4 involved in the shuttling of FFA in adipocytes and modulation of lipid metabolisms was reported to be linked with obesity and insulin resistance [29]–[32]. Although FABP4 is a well-known target of PPARγ in adipocytes [33], its increase in DDT knockdown adipocytes may be mainly due to suppressed expression of the AEBP1 gene, whose product is an FABP4 transcription repressor [34], because PPARγ mRNA levels were not significantly increased in DDT knockdown cells. Interestingly, mRNA levels of C/EBPα and leptin were increased in DDT knockdown cells. DDT knockdown adipocytes may show a phenotype similar to adipocytes in obese patients, because mRNA levels of C/EBPα and leptin, but not PPARγ, are reported to be higher in adipose tissues in obese subjects [35]. Down-regulation of DDT expression may have a role in altering the gene expression profile to that of obese adipocytes.

AMPK's inactivation followed by possible increased plasma FFA levels and the up-regulation of FABP4 expression in DDT knockdown adipocytes suggested reduced DDT expression in adipocytes to be associated with insulin resistance. Indeed, rDDT administration improved glucose tolerance in db/db mice, in which Ddt mRNA levels in the adipose tissue were reduced. Although we could not elucidate whether rDDT affects tissues other than adipose tissue, increased Ser-565 and decreased Ser-660 phosphorylation of HSL, resulting in its lower activity, and the decrease of FABP4 expression in the adipose tissue from rDDT-treated db/db mice suggest that the actions of DDT on adipocytes contribute to the improvement in glucose intolerance. Further studies are necessary to examine the involvement of DDT in other tissues such as liver and muscle tissue, that influence insulin sensitivity.

**Figure 7:**
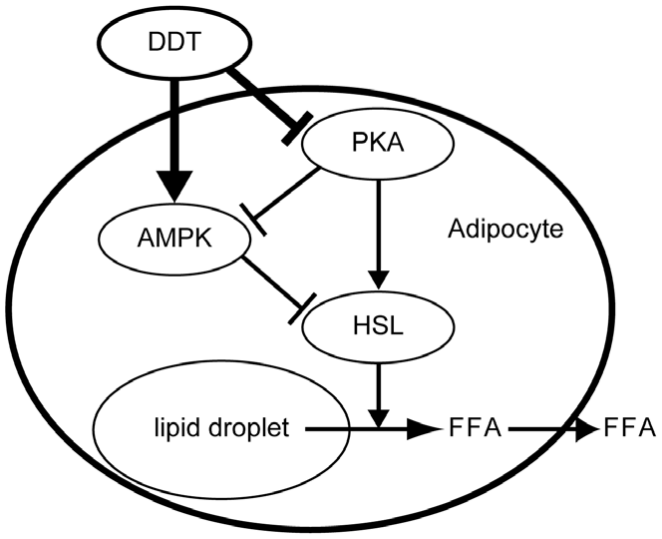
Inhibition of lipolysis by DDT in adipocytes. PKA inactivation by DDT brings about increased AMPK activity and decreased HSL activity. The AMPK activation induces further inhibition of HSL activity, followed by inhibiting hydrolysis of triacylglycerol to FFA that are then released from the adipocytes.

Plasma FFA levels are inversely associated with insulin sensitivity, especially in obese subjects [36], [37]. In the adipose tissue from rDDT-treated db/db mice, increased phosphorylation of the inactive type of HSL should decrease the levels of circulating FFA. Therefore, we postulated that inhibition of lipolysis in adipocytes followed by reduced plasma FFA levels is a mechanism of rDDT-induced insulin sensitivity in db/db mice. In adipocytes, elevated cellular cAMP production and PKA activated through the β-adrenergic receptor stimulate lipolysis by phosphorylating HSL directly or indirectly via AMPK [15], [38]. Basal PKA activity was reduced in the adipose tissue from rDDT-treated db/db mice, suggesting that DDT functions to limit lipolysis by inhibiting basal PKA activity (Fig. 7). However, rDDT did not affect basal levels of AMPKα and HSL phosphorylated by PKA in DDT knockdown adipocytes *in vitro*. This discrepancy may be due to difference of regulation of basal PKA activity between adipocytes *in vivo* and *in vitro*, because PKA activity in adipocytes *in vivo* is intricately regulated by sympathetic neurons or other factors. There is a possibility that DDT inhibits somewhere of these pathways regulating basal PKA activity. Otherwise, inhibition of basal PKA activity in the adipose tissue from db/db mice treated with DDT may be an indirect effect by other factors secreted from other tissues induced by rDDT. On the other hand, rDDT administration increased levels of phosphorylated AMPK at Thr-172 *in vitro* experiments in spite of no effects of phosphorylation by PKA, suggesting that DDT can also activate AMPK by a PKA-independent pathway. However, levels of phosphorylated AMPKα at Thr-172 did not show remarkable changes in adipose tissues from db/db mice. Basal AMPK activity in adipose tissue from db/db mice may be affected by endogenous DDT or other factors. In this study, we could not demonstrate the mechanism of the inactivation of PKA or activation of AMPK by DDT. These mechanisms and more detail function of DDT in adipocytes may be uncovered by developing DDT-knockout mice.

More recently, DDT was reported to be functional homolog of MIF because DDT binds CD74, a component of MIF receptor complex, leading to activation of ERK and its downstream proinflammatory pathways [10]; however, the expression pattern and function between MIF and DDT in adipose tissues may be different. For example, MIF is expressed not only adipocytes, but also preadipocytes where its mRNA levels do not increase with differentiation [39]. Moreover, serum MIF levels are elevated in patients with obesity and type 2 diabetes [40] and mRNA levels of MIF in subcutaneous abdominal adipose cells are positively associated with adipocyte size and insulin resistance [41]. MIF deficiency increases glucose uptake into the white adipose tissue [42] and improves insulin sensitivity and reduces macrophage infiltration in the white adipose tissue [43], suggesting that MIF brings about insulin resistance in adipose tissues. On the other hand, MIF was recently found to be released in the ischemic heart and stimulate the activation of AMPK [44], suggesting possibility that MIF stimulates AMPK in adipocytes as well as DDT. Further study will be needed to clarify the functional relationship between MIF and DDT in adipocytes.

In conclusion, DDT is a protein secreted from adipocytes and acts on adipocytes to regulate basal lipid metabolism by inhibiting the expression of genes involved in lipogenesis and lipolysis and by inhibiting activities of HSL and ACC. Notably, down-regulation of FABP4 expression and inhibition of lipolysis through AMPK activation and/or PKA inactivation in adipocytes may be involved in the improvement of obesity-associated glucose tolerance by DDT.

## References

[1] Rosen ED, Spiegelman BM (2006). Adipocytes as regulators of energy balance and glucose homeostasis.. Nature.

[2] Ronti T, Lupattelli G, Mannarino E (2006). The endocrine function of adipose tissue: an update.. Clin Endocrinol (Oxf).

[3] Odh G, Hindemith A, Rosengren AM, Rosengren E, Rorsman H (1993). Isolation of a new tautomerase monitored by the conversion of D-dopachrome to 5,6-dihydroxyindole.. Biochem Biophys Res Commun.

[4] Nishihira J, Fujinaga M, Kuriyama T, Suzuki M, Sugimoto H (1998). Molecular cloning of human D-dopachrome tautomerase cDNA: N-terminal proline is essential for enzyme activation.. Biochem Biophys Res Commun.

[5] Sugimoto H, Taniguchi M, Nakagawa A, Tanaka I, Suzuki M (1999). Crystal structure of human D-dopachrome tautomerase, a homologue of macrophage migration inhibitory factor, at 1.54 Å resolution.. Biochemistry.

[6] Bloom BR, Bennett B (1966). Mechanism of a reaction in vitro associated with delayed-type hypersensitivity.. Science.

[7] Calandra T, Bernhagen J, Mitchell RA, Bucala R (1994). The macrophage is an important and previously unrecognized source of macrophage migration inhibitory factor.. J Exp Med.

[8] Rosengren E, Bucala R, Åman P, Jacobsson L, Odh G (1996). The immunoregulatory mediator macrophage migration inhibitory factor (MIF) catalyzes a tautomerization reaction.. Mol Med.

[9] Coleman AM, Rendon BE, Zhao M, Qian MW, Bucala R (2008). Cooperative regulation of non-small cell lung carcinoma angiogenic potential by macrophage migration inhibitory factor and its homolog, D-dopachrome tautomerase.. J Immunol.

[10] Merk M, Zierow S, Leng L, Das R, Du X (2011). The D-dopachrome tautomerase (DDT) gene products a cytokine and functional homolog of macrophage migration inhibitory factor (MIF).. Proc Natl Acad Sci U S A.

[11] Guiherme A, Virbasius JV, Puri V, Czech MP (2008). Adipocyte dysfunctions linking obesity to insulin resistance and type 2 diabetes.. Nat Rev Mol Cell Biol.

[12] Hardie DG, Hawley SA, Scott JW (2006). AMP-activated protein kinase - development of the energy sensor concept.. J Physiol.

[13] Carling D, Sanders MJ, Woods A (2008). The regulation of AMP-activated protein kinase by upstream kinases.. Int J Obes (Lond).

[14] Hurley RL, Barré LK, Wood SD, Anderson KA, Kemp BE (2006). Regulation of AMP-activated protein kinase by multisite phosphorylation in response to agents that elevate cellular cAMP.. J Biol Chem.

[15] Djouder N, Tuerk RD, Suter M, Salvioni P, Thali RF (2010). PKA phosphorylates and inactivates AMPKα to promote efficient lipolysis.. EMBO J.

[16] Steinberg GR, Kemp BE (2009). AMPK in health and disease.. Physiol Rev.

[17] Assifi MM, Suchankova G, Constant S, Prentki M, Saha AK (2005). AMP-activated protein kinase and coordination of hepatic fatty acid metabolism of starved/carbohydrate-refed rats.. Am J Physiol Endocrinol Metab.

[18] Garton AJ, Yeaman SJ (1990). Identification and role of the basal phosphorylation site on hormone-sensitive lipase.. Eur J Biochem.

[19] Iwata T, Kuwajima M, Sukeno A, Ishimaru N, Hayashi Y (2009). YKL-40 secreted from adipose tissue inhibits degradation of type I collagen.. Biochem Biophys Res Commun.

[20] Wabitsch M, Brenner RE, Melzner I, Braun M, Möller P (2001). Characterization of a human preadipocyte cell strain with high capacity for adipose differentiation.. Int J Obes Relat Metab Disord.

[21] Sonesson B, Rosengren E, Hansson AS, Hansson C (2003). UVB-induced inflammation gives increased D-dopachrome tautomerase activity in blister fluid which correlates with macrophage migration inhibitory factor.. Exp Dermatol.

[22] Hiyoshi M, Konishi H, Uemura H, Matsuzaki H, Tsukamoto H (2009). D-Dopachrome tautomerase is a candidate for key proteins to protect the rat liver damaged by carbon tetrachloride.. Toxicology.

[23] Yang F, Yan S, He Y, Wang F, Song S (2008). Expression of hepatitis B virus proteins in transgenic mice alters lipid metabolism and induces oxidative stress in the liver.. J Hepatol.

[24] Strey CW, Winters MS, Markiewski MM, Lambris JD (2005). Partial hepatectomy induced liver proteome changes in mice.. Proteomics.

[25] Woods A, Azzout-Marniche D, Foretz M, Stein SC, Lemarchand P (2000). Characterization of the role of AMP-activated protein kinase in the regulation of glucose-activated gene expression using constitutively active and dominant negative forms of the kinase.. Mol Cell Biol.

[26] Zhou G, Myers R, Li Y, Chen Y, Shen X (2001). Role of AMP-activated protein kinase in mechanism of metformin action.. J Clin Invest.

[27] Minokoshi Y, Kim YB, Peroni OD, Fryer LG, Müller C (2002). Leptin stimulates fatty-acid oxidation by activating AMP-activated protein kinase.. Nature.

[28] Yamauchi T, Kamon J, Minokoshi Y, Ito Y, Waki H (2002). Adiponectin stimulates glucose utilization and fatty-acid oxidation by activating AMP-activated protein kinase.. Nat Med.

[29] Hertzel AV, Bernlohr DA (2000). The mammalian fatty acid-binding protein multigene family: molecular and genetic insights into function.. Trends Endocrinol Metab.

[30] Hotamisligil GS, Johnson RS, Distel RJ, Ellis R, Papaioannou VE (1996). Uncoupling of obesity from insulin resistance through a targeted mutation in aP2, the adipocyte fatty acid binding protein.. Science.

[31] Uysal KT, Scheja L, Wiesbrock SM, Bonner-Weir S, Hotamisligil GS (2000). Improved glucose and lipid metabolism in genetically obese mice lacking aP2.. Endocrinology.

[32] Makowski L, Boord JB, Maeda K, Babaev VR, Uysal KT (2001). Lack of macrophage fatty-acid-binding protein aP2 protects mice deficient in apolipoprotein E against atherosclerosis.. Nat Med.

[33] Tontonoz P, Nagy L, Alvarez JG, Thomazy VA, Evans RM (1998). PPARγ promotes monocyte/macrophage differentiation and uptake of oxidized LDL.. Cell.

[34] He GP, Muise A, Li AW, Ro HS (1995). A eukaryotic transcriptional repressor with carboxypeptidase activity.. Nature.

[35] Krempler F, Breban D, Oberkofler H, Esterbauer H, Hell E (2000). Leptin, peroxisome proliferator-activated receptor-γ, and CCAAT/enhancer binding protein-α mRNA expression in adipose tissue of humans and their relation to cardiovascular risk factors.. Arterioscler Thromb Vasc Biol.

[36] Unger RH (1995). Lipotoxicity in the pathogenesis of obesity-dependent NIDDM genetic and clinical implications.. Diabetes.

[37] Guilherme A, Virbasius JV, Czech MP (2008). Adipocyte dysfunction linking obesity to insulin resistance and type 2 diabetes.. Nat Rev Mol Cell Biol.

[38] Londos C, Brasaemle DL, Schultz CJ, Adler-Wailes DC, Levin DM (1999). On control of lipolysis in adipocytes.. Ann NY Acad Sci.

[39] Skurk T, Herder C, Kräft I, Müller-Scholze S, Hauner H (2005). Production and release of macrophage migration inhibitory factor from human adipocytes.. Endocrinology.

[40] Kleemann R, Bucala R (2010). Macrophage migration inhibitory factor: critical role in obesity, insulin resistance, and associated comorbidities.. Mediators Inflamm.

[41] Koska J, Stefan N, Dubois S, Trinidad C, Considine RV (2009). mRNA concentrations of MIF in subcutaneous abdominal adipose cells are associated with adipocyte size and insulin action.. Int J Obes (Lond).

[42] Atsumi T, Cho YR, Leng L, McDonald C, Yu T (2007). The proinflammatory cytokine macrophage migration inhibitory factor regulates glucose metabolism during systemic inflammation.. J Immunol.

[43] Verschuren L, Kooistra T, Bernhagen J, Voshol PJ, Ouwens DM (2009). MIF deficiency reduces chronic inflammation in white adipose tissue and impairs the development of insulin resistance, glucose intolerance, and associated atherosclerotic disease.. Circ Res.

[44] Miller EJ, Li J, Leng L, McDonald C, Atsumi T (2008). Macrophage migration inhibitory factor stimulates AMP-activated protein kinase in the ischaemic heart.. Nature.

